# Mitotic UV Irradiation Induces a DNA Replication-Licensing Defect that Potentiates G1 Arrest Response

**DOI:** 10.1371/journal.pone.0120553

**Published:** 2015-03-23

**Authors:** Masayuki Morino, Kohei Nukina, Hiroki Sakaguchi, Takeshi Maeda, Michiyo Takahara, Yasushi Shiomi, Hideo Nishitani

**Affiliations:** Graduate School of Life Science, University of Hyogo, Kamigori, Ako-gun, Hyogo, Japan; Florida State University, UNITED STATES

## Abstract

Cdt1 begins to accumulate in M phase and has a key role in establishing replication licensing at the end of mitosis or in early G1 phase. Treatments that damage the DNA of cells, such as UV irradiation, induce Cdt1 degradation through PCNA-dependent CRL4-Cdt2 ubiquitin ligase. How Cdt1 degradation is linked to cell cycle progression, however, remains unclear. In G1 phase, when licensing is established, UV irradiation leads to Cdt1 degradation, but has little effect on the licensing state. In M phase, however, UV irradiation does not induce Cdt1 degradation. When mitotic UV-irradiated cells were released into G1 phase, Cdt1 was degraded before licensing was established. Thus, these cells exhibited both defective licensing and G1 cell cycle arrest. The frequency of G1 arrest increased in cells expressing extra copies of Cdt2, and thus in cells in which Cdt1 degradation was enhanced, whereas the frequency of G1 arrest was reduced in cell expressing an extra copy of Cdt1. The G1 arrest response of cells irradiated in mitosis was important for cell survival by preventing the induction of apoptosis. Based on these observations, we propose that mammalian cells have a DNA replication-licensing checkpoint response to DNA damage induced during mitosis.

## Introduction

Proper progression of the cell cycle depends on the periodic activation of cyclin-dependent protein kinases (CDKs) [1]. To initiate DNA replication, replication origins are “licensed” for replication by the formation of a pre-replicative complex in late M phase or early G1 phase. Licensing is achieved when the complex of minichromosome maintenance proteins 2–7 (MCM2-7), with the help of Cdc6 and Cdt1, is loaded onto sites bound by the origin-recognition complex [2,3,4]. Activation of the replication kinases S-CDK and DDK triggers the firing of licensed origins for one round of DNA replication [5]. Among the licensing factors, Cdt1 levels are strictly regulated in mammalian cells. Cdt1 begins accumulating during M phase with levels peaking in G1 phase, but it is degraded and maintained at a low level once DNA replication is initiated. Such regulation is important for preventing the re-replication of chromosomes [4,6,7]. In mammalian cells, pathways mediated by two Cullin-ring finger ubiquitin ligases, CRL1^Skp2^ (also known as SCF-Skp2) and CRL4^Cdt2^ (also known as Cul4-DDB1-Cdt2), operate independently to degrade Cdt1 [8,9,10,11,12]. Cdt2 is a WD40 repeat-containing protein isolated as a damage-specific DNA-binding protein 1 (DDB1) that acts as a substrate receptor protein [13,14,15]. Importantly, Cdt1 has a specialized motif for destruction at the N-terminus, called the PIP-degron, which comprises the PIP-box, TD amino acids, and basic amino acids (Q-[V/I/L/M]-T-D-[F/Y]-[F/Y]-x-x-B-B)[16,17]. Cdt1 binds to proliferating cell nuclear antigen (PCNA) through the PIP box and the resulting PIP-degron exposed on the PCNA is recognized by CRL4^Cdt2^[18]. Thus, when DNA replication is initiated, PCNA connects Cdt1 and CRL4^Cdt2^ on the chromatin for ubiquitination, thereby preventing illegal re-replication.

To maintain genome integrity, cells must be also able to respond to genotoxic insults by triggering DNA-damage responses, including DNA damage-induced checkpoint activation and DNA repair [19,20]. Ultraviolet (UV) irradiation induces helix-distorting DNA lesions, such as cyclobutane pyrimidine dimers (CPDs) and 6–4 photoproducts, on genomic DNA. Nucleotide excision repair (NER) is a versatile system for repairing UV-induced DNA lesions [21,22,23,24]. UV-induced DNA damage is recognized by CRL4^DDB2^, which binds to CPDs and 6–4 photoproducts, and ubiquitinates xeroderma pigmentosum complementation group C protein and DDB2 to initiate NER. Cells with a DDB2 mutation are classified as a xeroderma pigmentosum complementation group E protein. Interestingly, Cdt1 is degraded after UV irradiation by the above-mentioned PCNA-mediated CRL4^Cdt2^ pathway [25,26,27,28]. Both Cdt1 and Cdt2-CRL4 were recruited to DNA damage sites marked by CPD or PCNA. Cdt1 requires its PIP-box for recruitment. During NER, a damage-containing strand is excised, and a single strand gap is created. PCNA loaded by replication factor C proteins, RFC1-RFC, at such a gap appears to recruit Cdt1 and CRL4^Cdt2^ for Cdt1 degradation.

In addition to UV irradiation, many DNA damaging reagents induce Cdt1 degradation [29,30,31]. How Cdt1 degradation is connected to the DNA damage response, however, is unclear. Here, we examined Cdt1 degradation after UV irradiation during different phases of the cell cycle. Mitotic cells were resistant to degradation after UV-irradiation, but when these cells were released into G1 phase, Cdt1 was degraded, and DNA replication licensing was severely inhibited. Such cells had a high frequency of G1 cell-cycle arrest. Our data suggested that in addition to the well-known DNA damage checkpoint response, cells have a replication licensing checkpoint that links mitotic DNA damage to cell cycle control.

## Results

### UV irradiation causes Cdt1 degradation in G1, but MCM2-7 proteins remains stable on chromatin

**Fig 1:**
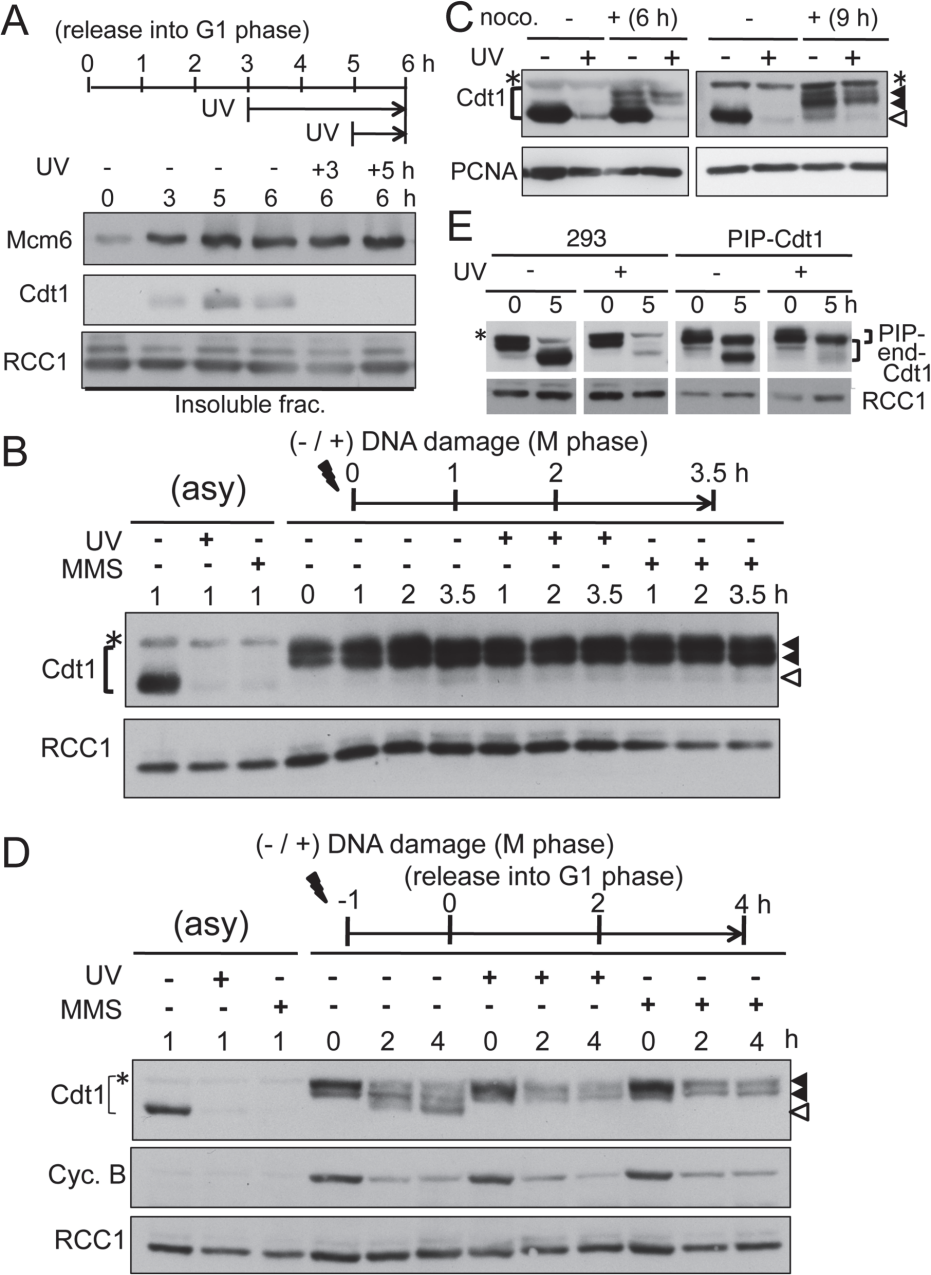
UV irradiation induces Cdt1 degradation in G1 phase, but not in mitosis. A. Mcm6 remains associated with chromatin after UV irradiation in G1 phase. Half of the dishes of mitotic HeLa cells, prepared as described in Materials and Methods, were released into G1 phase and collected at the indicated time-points and the other half of the dishes of cells were UV-irradiated at 3 h (+3) or 5 h (+5) after release and collected at 6 h. Insoluble fractions were prepared after centrifuging the cell lysates and blotted with the indicated antibodies. RCC1 was used as a control. B. Mitotic HeLa cells (M phase) were incubated without any treatment (-), irradiated with UV (50 J/m^2^) and incubated (+), or incubated in the presence of MMS (1 mM) (+) for the indicated time (h). Cells were collected, and whole cell extracts were made and blotted with the indicated antibodies. Asynchronously growing cells (asy) treated similarly (+) or not (-) and collected 1 h later were included as a control. Closed arrow-heads indicate the hyperphosphorylated forms of Cdt1, and open arrowhead indicates the fast migrating form of G1 phase. * indicates bands not specific for Cdt1. C. Asynchronously growing HeLa cells were treated with nocodazole at 40 ng/ml (+) for 6 or 9 h or not (-), irradiated with UV (50 J/m^2^) and incubated for 1 h. Whole cell extracts were prepared and blotted with the indicated antibodies. PCNA was used as a loading control. D. Mitotic HeLa cell cultures (M phase) were UV-irradiated (50 J/m^2^) (+), treated with MMS (1 mM) (+) or not (-) (-1 h), and incubated for1 h, then cells were washed out of nocodazole (and MMS) for release into G1 phase for 0, 2, and 4 h. Whole cell extracts were made and blotted with the indicated antibodies. Cyclin B was used to monitor the exit from mitosis (Cyc.B). Asynchronously growing cells (asy) treated similarly (+) or not (-) and collected 1 h later were included as a control. E. Control or PIP-box mutated Cdt1 (PIP-Cdt1:A6-Cdt1-3NLSmyc) expressing HEK293 cells were synchronized in M phase, UV-irradiated (40 J/m^2^) (+) or not (-), and released into G1 phase. Cells were collected at time 0 and 5 h, whole cell extracts were prepared and blotted with the indicated antibodies. Endogenous Cdt1 (end-.) and PIP-box mutated Cdt1 (PIP-) were indicated.

**Fig 2:**
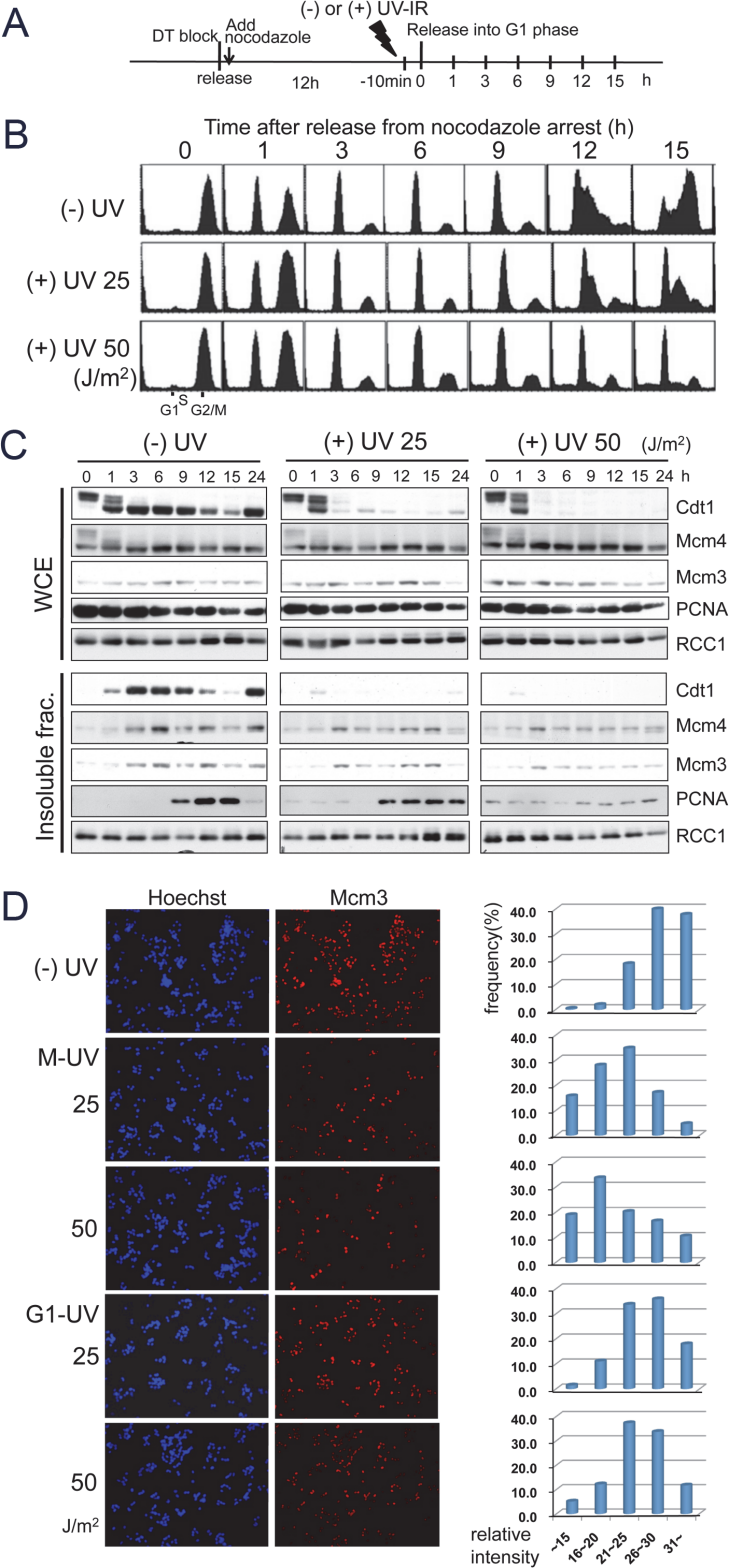
UV-irradiated M phase-cells show decreased MCM 2–7 loading and G1 arrest after release: HeLa cells. A. Scheme of synchronization and sample preparation. DT block: double thymidine block. B and C. HeLa cells synchronized in M phase were released without UV irradiation [(-)UV], or UV-irradiated at 25 J/m^2^ or 50 J/m^2^ [(+)UV] and released 10 min later. Cells were collected at the indicated time-points for flow cytometry (B) and for preparation of whole cell extracts (WCE) and insoluble fractions for immunoblotting (C). D. Immunofluorescent analysis of MCM2-7 loading. Cells released without UV irradiation [(-)UV] or after UV irradiation in M phase (M-UV) or in G1 phase at 3 h after release (G1-UV) were pre-extracted and fixed at 8 h and stained for Mcm3. Mcm3 signals for each cell were measured and the signal intensity distribution profile was plotted.

Because UV irradiation induces Cdt1 degradation, we investigated how DNA replication licensing is affected by examining chromatin associated MCM2-7. DNA replication licensing takes place by early G1 phase and thus most G1 cells already have MCM2-7 loaded on the chromatin. Thus, it was expected that MCM2-7 was present on chromatin even after Cdt1 was degraded. We confirmed this notion using a synchronized HeLa cell culture made by release from nocodazole-induced mitotic arrest into G1 phase. At 3 to 5 h after release, the cells were traversing G1 phase and Mcm6, a component of the MCM2-7 complex, was loaded on the chromatin at high levels (Fig. 1A: see synchronization profile of HeLa cells in Fig. 2). We irradiated the HeLa cells with UV at these time-points in G1 phase (we named these cells HeLa [G1-UVIR] cells) and collected them at 6 h after release. Although Cdt1 was degraded, the Mcm6 levels on the chromatin in UV-irradiated cells were similar to those in non-irradiated cells (Fig. 1A). These observations suggested that once MCM2-7 was loaded onto the chromatin, MCM2-7 remained rather stable on the chromatin even if Cdt1 was degraded after exposure to UV in G1 phase.

### Cdt1 was not degraded in M phase after UV-irradiation, but was degraded when released into G1 phase

We then examined how cells respond to UV-irradiation in M phase. Hyperphosphorylated Cdt1 was present in M phase, as we previously reported [6]. Interestingly, in contrast to G1 phase, Cdt1 in mitotic cells was not degraded and remained stable for more than 3 h post irradiation. To see whether this response was specific to UV-irradiation, we examined cells exposed to methyl methane sulfonate (MMS), a drug that produces alkylated DNA damage. As previously shown[30], MMS treatment induced Cdt1 degradation in asynchronously growing cells within 1 h. Similar to the UV irradiation, MMS treatment did not induce Cdt1 degradation in mitotic cells (Fig. 1B). The defect in Cdt1 degradation was not due to the effect of nocodazole, because nocodazole treatment did not inhibit UV-induced Cdt1 degradation in G1 phase cells (Fig. 1C). When asynchronously growing cells were treated with nocodazole for 6 or 9 h, fast migrating forms and hyperphosphorylated forms of Cdt1 were detected, which represent Cdt1 in G1 phase and in M phase, respectively. The Cdt1 in G1 phase was degraded even in the presence of nocodazole.

We then investigated how damaged mitotic cells respond when released into G1 phase. After washing out the nocodazole, the control cells (- UV and - MMS) moved into G1 phase and dephosphorylated forms of Cdt1 accumulated in the cells (Fig. 1D), as we previously reported[6]. In contrast, when UV-irradiated cells were released, the Cdt1 was degraded as the cells progressed into G1 phase (Fig. 1D). A similar response was observed in MMS-treated cells. The decrease in Cyclin B levels verified that the kinetics of the exit from mitosis were not affected by UV or MMS treatment. Degradation of Cdt1 after treatment with DNA-damaging agents was dependent on CRL4^Cdt2^ ubiquitin ligase. To confirm that this ligase was involved in the degradation of Cdt1 as the cells moved into G1 phase, we used HEK293 cells expressing Cdt1 mutated at the PIP-box and tagged with 3NLSmyc (A6-Cdt1-3NLSmyc), which cannot associate with PCNA and thus cannot be targeted by CRL4^Cdt2^ [8]. Cells expressing PIP-box mutated Cdt1 were released into G1 phase. Although endogenous Cdt1 was degraded similar to control HEK293 cells, the PIP-box mutated Cdt1 remained stable after release into G1 phase (Fig. 1E). Taken together, these observations indicated that Cdt1 in M phase was resistant to DNA damage-induced degradation, but was degraded by the CRL4^Cdt2^ pathway as the damaged mitotic cells moved into G1 phase.

### Mitotic cells irradiated with UV were defective in MCM2-7 loading when they entered G1 phase

The above data suggested that Cdt1 was degraded at very early stage in G1 phase after release. Therefore, we wondered if cells irradiated during M phase are defective in replication licensing for the ensuing cell cycle. To clarify this notion, we irradiated cells with different doses of UV during mitotic arrest, released them from mitosis, and then examined licensing states and cell cycle progression (Fig. 2A). When non-irradiated control cells were released from arrest, Cdt1 was dephosphorylated and associated with chromatin, peaking around 3 h after release, followed by the association of Mcm3 and Mcm4 with chromatin, which peaked around 6 h (Fig. 2C). Subsequently, the cells entered S phase around 9 h after release, as revealed by flow cytometry analysis, and by an increase in the PCNA-chromatin association (Fig. 2B and 2C). Consistent with these observations, Cdt1 degradation started around 9–12 h. Another culture of nocodazole-arrested cells was irradiated with a dose of 25 J/m^2^ or 50 J/m^2^ (named HeLa [M-UVIR] cells) and released from arrest 10 min later. Although the kinetics of the exit from mitosis and entry into G1 phase were not affected by UV irradiation (Fig. 2B), Cdt1 was degraded around 3 h after release, just when cells entered into G1 phase, and chromatin-associated Cdt1 was hardly detected (Fig. 2C). In accordance with Cdt1 degradation, the chromatin association of Mcm3 and Mcm4 was severely inhibited. The Cdt1 degradation and MCM-loading inhibition were more prominent in cells irradiated with the higher dose (50 J/m^2^). Because Cdt1 degradation is dependent on chromatin-loaded PCNA, we investigated PCNA levels on the chromatin. In control cells (- UV), PCNA was not detected on chromatin in M phase cells (Fig. 2C, 0 h). In contrast, although the levels were lower than that seen in S phase of control cells, PCNA was detected on chromatin at time 0 h, 10 min after UV-irradiation and thereafter in UV-irradiated cells, and at higher levels in 50 J/m^2^ irradiated cells than in 25 J/m^2^ irradiated cells. In cells irradiated at 50 J/m^2^, the PCNA levels on chromatin appeared to be remained the same thereafter, while the levels in cells irradiated at 25 J/m^2^ increased around 9 h, when a population of cells entered S phase (see below). In UV irradiated cells (both at 25 J/m^2^ and at 50 J/m^2^), Cdt1 was degraded at 3 h. Thus, we speculate that the loading of PCNA started during M phase after UV-irradiation, but Cdt1 degradation was inhibited. As cells moved into G1 phase, the cells became competent to use chromatin-bound PCNA for Cdt1 degradation (see Discussion).

Immunofluorescence analysis of individual cells confirmed that the association of MCM2-7 with chromatin was reduced in HeLa [M-UVIR] cells (Fig. 2D). Chromatin-associated MCM2-7 proteins were detected by fixing the cells after pre-extraction with detergent to remove the chromatin-unbound fractions [32]. At 8 h after release from mitotic arrest, most of non-irradiated cells were stained with Mcm3, revealing that the cells were licensed for DNA replication. The frequency of Mcm3-positive cells was reduced to around 25% in HeLa [M-UVIR] cells. In contrast, almost all HeLa [G1-UVIR] cells irradiated at 3 h after release, were stained with Mcm3 at levels similar to those of non-irradiated cells, consistent with the immunoblotting assay (Fig. 1A). We also measured the nuclear Mcm3 signals of individual cells and plotted the distribution of their signal intensity. Although HeLa [G1-UVIR] cells had high intensity Mcm3-staining, most of the HeLa [M-UVIR] cells had smaller signals (Fig. 2D right).

These findings suggested that while Cdt1 was stable in M phase after UV irradiation, Cdt1 was degraded as the cells exited from M phase, and replication licensing was inhibited.

### Cells exposed to UV during mitosis are arrested in G1 phase

While control HeLa cells entered S phase around 9 h after release, a large population of HeLa [M-UVIR] cells was defective for entry into S phase (Fig. 2B). At a higher dose of UV, 50 J/m^2^, most irradiated cells arrested in G1 phase. Consistent with the inhibition of entry into S phase, the association of PCNA on the chromatin around 9 h onwards was greatly reduced in these cells (Fig. 2C). These results suggested that HeLa [M-UVIR] cells displayed both a licensing defect and a G1 arrest response.

**Fig 3:**
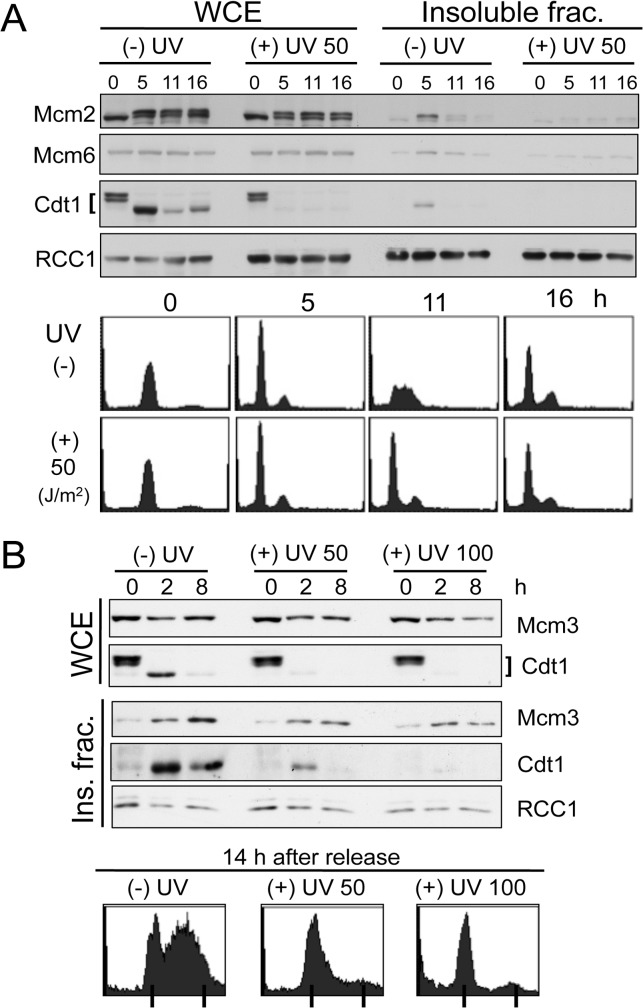
UV-irradiated M-phase cells show decreased MCM 2–7 loading and G1 arrest after release: HEK293 cells and U2OS cells. A. HEK293 cells synchronized in M phase were released without UV irradiation [(-)UV], or UV-irradiated with 50 J/m^2^ [(+)UV] and released 10 min later. Cells were collected and treated as described in Fig. 2B and 2C. WCE, whole cell extract. B. U2OS cells UV-irradiated in M phase and released. U2OS cells synchronized in M phase were released or UV-irradiated at 50 J/m^2^ or 100 J/m^2^ and released 10 min later. Cells were collected and treated as described in Fig. 2B and 2C.

To examine whether other cell lines would respond similarly to mitotic UV irradiation, we synchronized HEK293 and U2OS cells in mitosis and analyzed their response to UV irradiation. Half of each culture was irradiated with UV, and released. Cdt1 was also detected in its highly phosphorylated forms in M phase in both of these cell lines (Fig. 3A and 3B). At 11 h after release, the non-irradiated HEK293 cells were in S phase, as revealed by the flow cytometry analysis. In the HEK293 [M-UVIR] cells, Cdt1 was degraded as the cells entered G1 phase, similar to HeLa [M-UVIR] cells. MCM2-7 loading was inhibited and most of cells were in G1 arrest (Fig. 3A). U2OS cells, which have normal p53 gene, also responded similarly to mitotic UV irradiation (Fig. 3B). These results demonstrated that UV irradiation of various cells in M phase causes a licensing defect and G1 cell-cycle arrest.

### Cells irradiated in M phase have a greater G1 arrest response than cells irradiated in G1 phase

**Fig 4:**
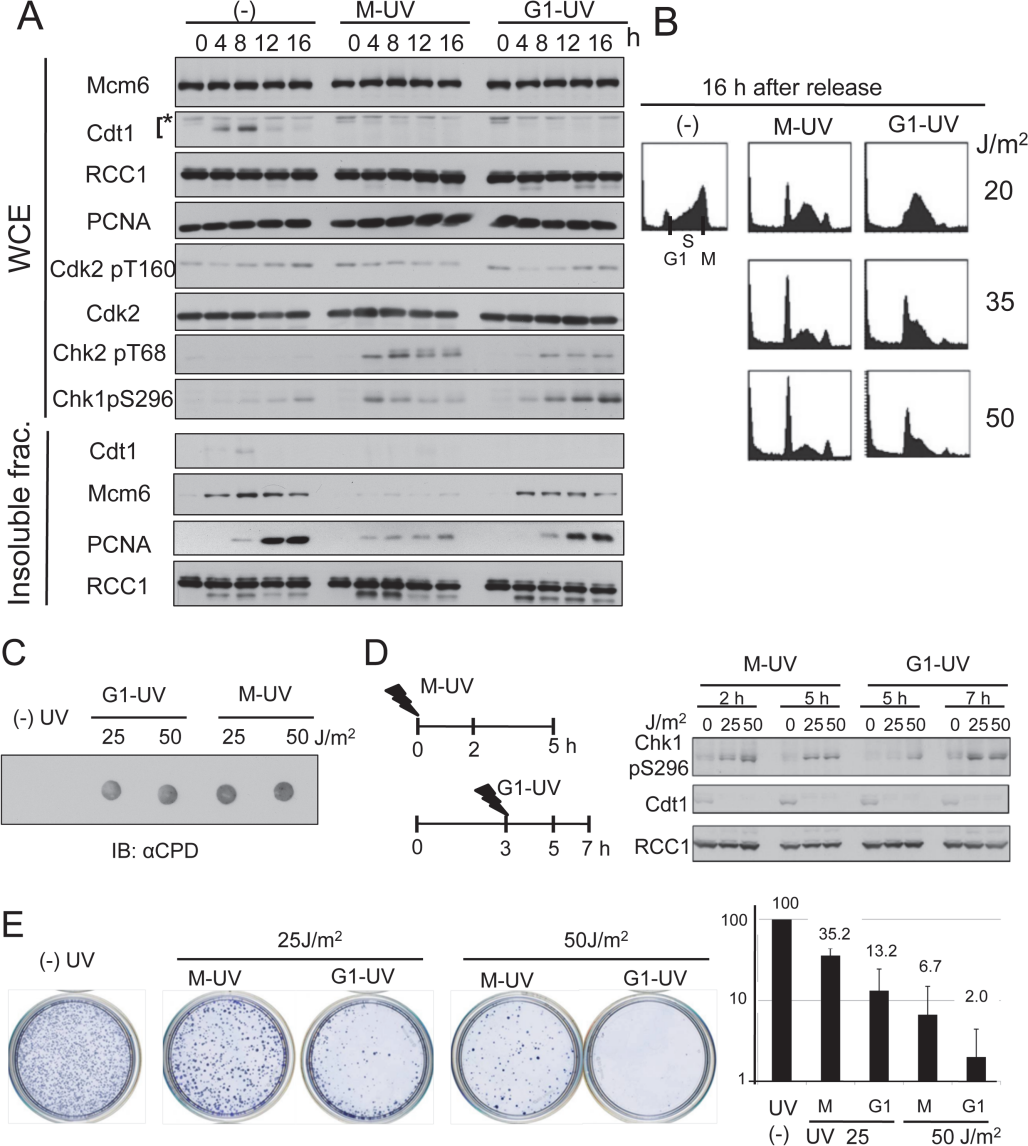
Cells released after UV irradiation in M phase show a stronger G1 arrest phenotype than cells UV-irradiated in G1 phase. A. Cdt1 degradation and chromatin association of proteins. HeLa cells synchronized in M phase were released (-), UV-irradiated at 40 J/m^2^, and released 10 min later (M-UV) or UV-irradiated after release at 3 h (G1-UV), and collected at the indicated time-points for the preparation of whole cell extracts (WCE) and an insoluble fraction. The indicated proteins were examined by Western blotting. B. Flow cytometry analysis of cells UV-irradiated during M phase or in G1 phase. Cells treated as in A were collected at 16 h after release for flow cytometry. C. Levels of CPD. Cells UV-irradiated in M phase or G1 phase at 3 h after release were measured for CPD levels by dot blotting. D. Levels of checkpoint activation. Cells UV-irradiated in M phase or in G1 phase at 3 h after release and collected at the indicated time-points for measurement of the P-S296 levels. E. Sensitivity of cells to UV irradiation in M phase or G1 phase. Cells UV-irradiated in M phase or G1 phase at 3 h after release were cultured for 2 weeks, and colony formation was measured. Triplicate experiments were performed. The colony numbers were normalized, set to 100% for UV(-) cells.

We then compared the cell cycle progression between HeLa cells irradiated in G1 phase [G1-UVIR] and those irradiated in M phase [M-UVIR]. For analysis of HeLa [G1-UVIR] cells, the cells were irradiated 3 h after release from M phase. Consistent with the previous observations (Figs. 1A and 2C), MCM2-7 levels on the chromatin in HeLa [G1-UVIR] were comparable with those in non-irradiated cells, while those in HeLa [M-UVIR] cells were reduced (Fig. 4A).

Because UV irradiation in the M phase or G1 phase of the cell cycle resulted in different levels of replication licensing, we investigated the cell-cycle progression of UV-irradiated cells. At 16 h after release from M phase, most of the non-irradiated control cells (- UV) were in late S phase as revealed by flow cytometry (Fig. 4B), while HeLa [M-UVIR] cells were arrested in G1. The frequency of G1-arrested cells increased with increasing UV irradiation dose. In contrast to HeLa [M-UVIR] cells, HeLa [G1-UVIR] cells had a lower frequency of G1 arrest at all UV irradiation doses examined. These results were also reflected in the levels of chromatin-associated PCNA. In non-irradiated cells (-), the chromatin-associated PCNA levels increased at 12 h and 16 h after release, as the cells were in S phase (Fig. 4A). In the case of HeLa [M-UVIR] cells, low levels of chromatin-associated PCNA was detected from the early time points but no increase was observed around at 12 h and 16 h after release, consistent with that of HeLa [M-UVIR] cells arrested in G1 phase. In contrast, chromatin-associated PCNA in HeLa [G1-UVIR] cells was detected at half the level of non-irradiated cells as they entered S phase. The RCC1 on the chromatin was detected at the same levels, irrespective of UV irradiation throughout the time-course analysis.

We wondered whether the difference in the extent of the G1 arrest response between HeLa [M-UVIR] cells and HeLa [G1-UVIR] cells was due to a difference in the levels of UV-induced damage. We isolated the chromosomal DNA after UV irradiation, and immunoblotted with anti-CPD antibody. No apparent difference in CPD levels was observed between HeLa [G1-UVIR] and [M-UVIR] cells (Fig. 4C). The levels of Chk1 activation were also almost the same between HeLa [G1-UVIR] and [M-UVIR] cells at 2 h or 4 to 5 h after UV-irradiation, when the cells were in G1 phase (Fig. 4D). Upon further incubation, higher levels of Chk1 activation were observed in HeLa [G1-UVIR] cells at 12 and 16 h (Fig. 4A). This was probably due to activation of the DNA replication checkpoint, as a large population of HeLa [G1-UVIR] cells entered S phase in the presence of DNA damage and DNA replication might have been blocked to activate checkpoint kinases. In these conditions, the cells would have undergone replication stress. If this were the case, HeLa [G1-UVIR] cells would be expected to be more sensitive to UV irradiation than HeLa [M-UVIR] cells. To examine this possibility, we performed a survival assay. HeLa [M-UVIR] and HeLa [G1-UVIR] cells irradiated at 25 J/m^2^ or 50 J/m^2^ were cultured for 2 weeks, and the colonies were counted. At both irradiation doses, the HeLa [M-UVIR] cells formed more colonies, suggesting that they were more resistant to UV irradiation than the HeLa [G1-UVIR] cells (Fig. 4E).

### Extra expression of Cdt2 enhances the G1 arrest response of cells irradiated in mitosis

**Fig 5:**
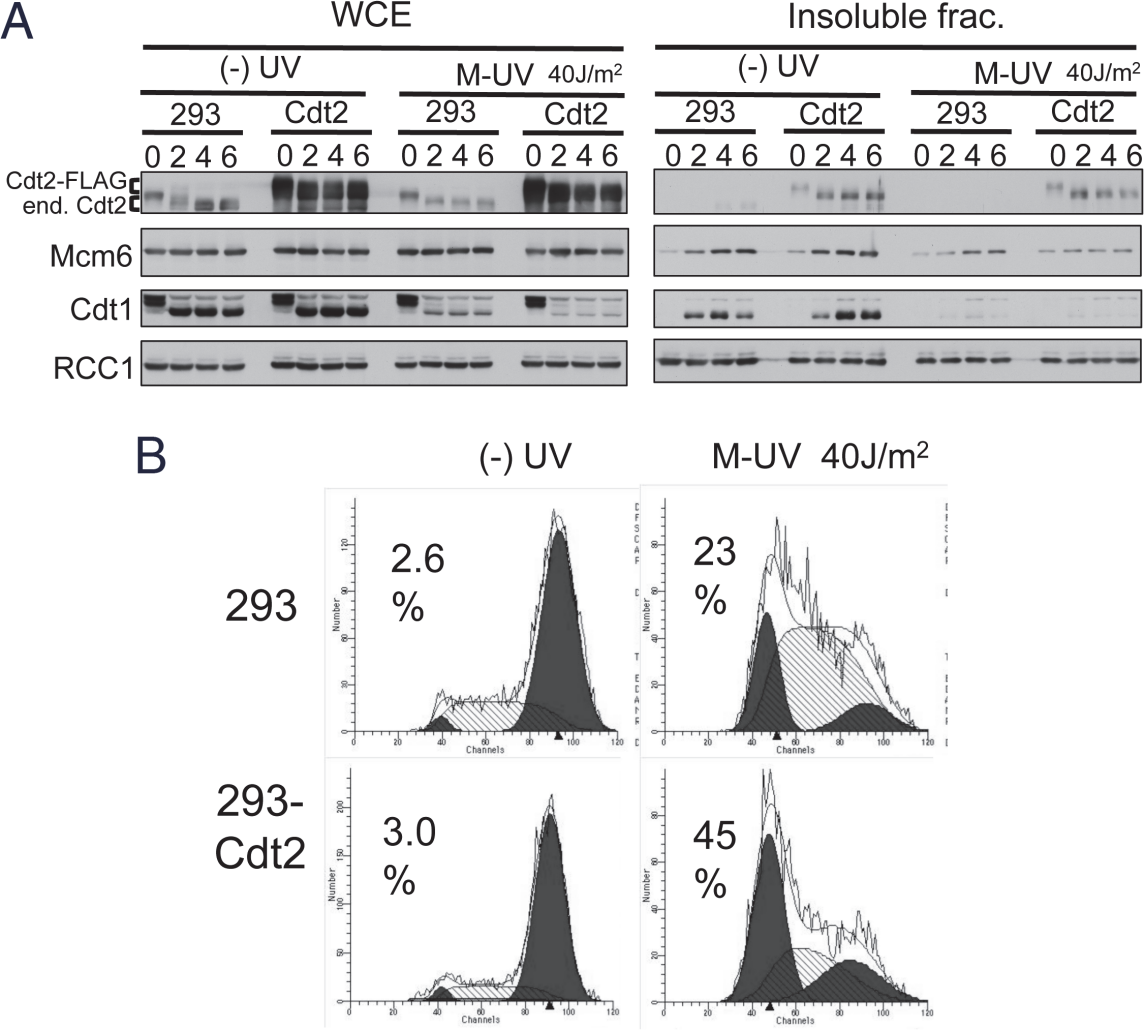
Ectopic expression of Cdt2 enhances the G1 arrest response. A. Cdt1 degradation and MCM2-7 loading in control HEK293 cells and Cdt2-FLAG expressing HEK293 cells. Control (293) and Cdt2-FLAG expressing HEK293 cells (Cdt2) were arrested in M phase and released with or without UV irradiation (40 J/m^2^). Cells were collected at the indicated time-points to prepare whole cell extracts (WCE) and an insoluble fraction. Note that Cdt2 (both endogenous (end.) and FLAG-tagged (Cdt2-FLAG)) were hyperphosphorylated in M phase[25]. B. Flow cytometry analysis of Cdt2-FLAG expressing cells. Cells treated as in A were collected 24 h after release for flow cytometry. To block cells in mitosis, nocodazole was added at the 10 h time-point. Frequency of G1 phase cells was shown (%).

The above observations suggested that although the DNA damage checkpoint was activated both in HeLa [M-UVIR] and HeLa [G1-UVIR] cells, additional control was involved in the G1 arrest response of HeLa [M-UVIR] cells. A licensing checkpoint response was previously reported in normal cells depleted of Cdt1 or Cdc6 by small interfering RNAs [33,34]. These licensing-defective normal cells were prevented from entering into S phase. If the licensing defect contributed to the G1 arrest of M-UVIR cells, we expected that the more complete the Cdt1 degradation, the greater the cell population in G1 arrest. To investigate this, we used a stable HEK293 cell line expressing an extra-copy of FLAG-tagged Cdt2, HEK293-Cdt2-FLAG (Fig. 5A). When both HEK293 cells and HEK293-Cdt2-FLAG cells were arrested in M phase and released without UV irradiation, Mcm6 was loaded on the chromatin with similar kinetics in both cell lines. On the other hand, when the cells were UV-irradiated during M phase and released, Cdt1 was more completely degraded and Mcm6 chromatin levels were reduced in HEK293-Cdt2-FLAG cells. Flow cytometry analysis demonstrated that HEK293-Cdt2-FLAG cells had a larger population of G1-arrested cells than control HEK293 cells (Fig. 5B), suggesting that a stronger licensing defect led to a greater G1 arrest response.

### Ectopic expression of Cdt1 reverses G1 arrest of mitotically UV-irradiated cells

**Fig 6:**
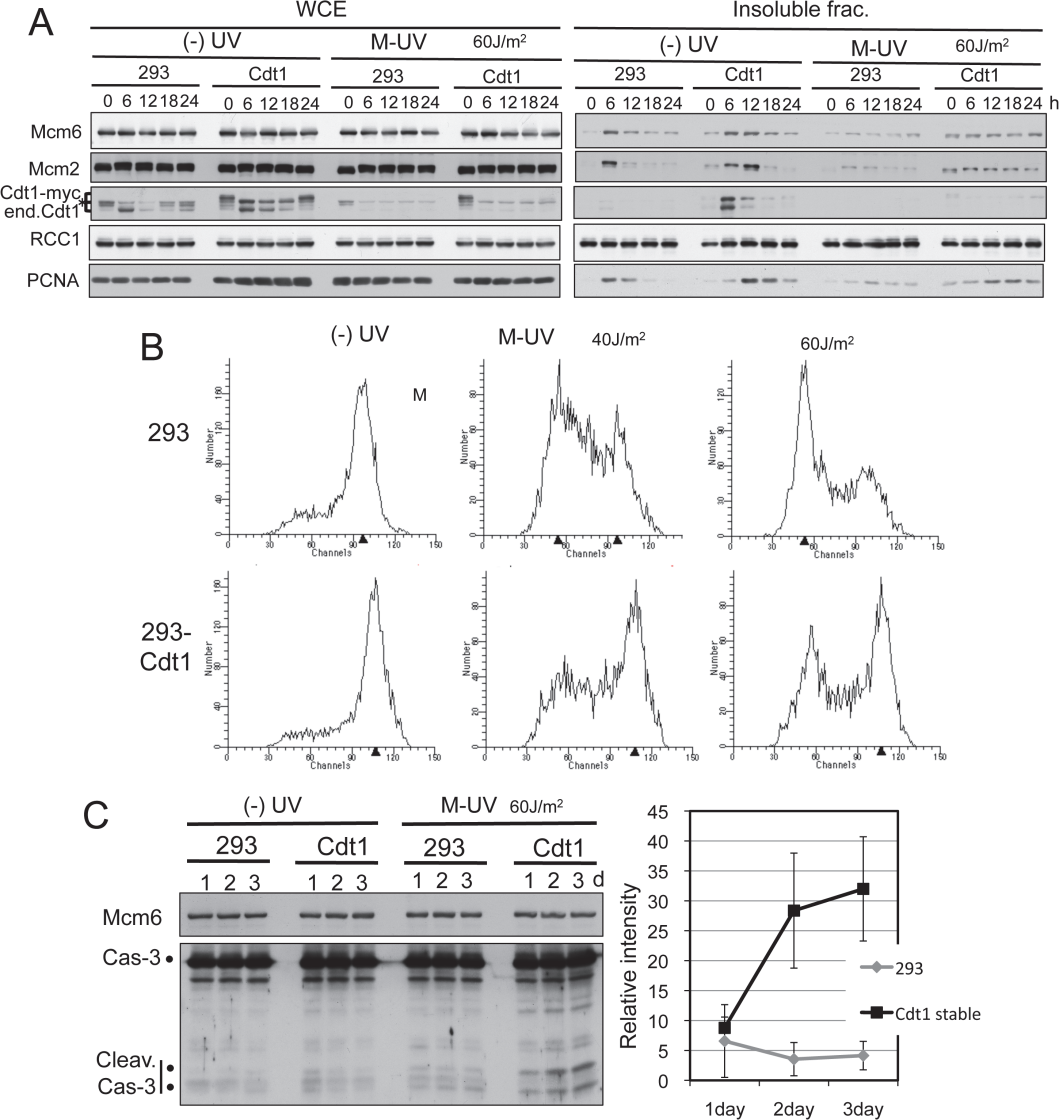
Ectopic expression of Cdt1 lowers the G1 arrest response. A. Extra copy of Cdt1 expressing cells have more MCM2-7 on the chromatin. Control (293) and Cdt1-3NLSmyc expressing HEK293 cells (Cdt1) were arrested in M phase and released with (M-UV) or without [(-)UV] UV irradiation (60 J/m^2^). Each cell line was collected at the indicated time-points for preparation of whole cell extracts (WCE) and an insoluble fraction. B. Flow cytometry analysis of Cdt1-3NLSmyc expressing cells. Cells treated as in A were collected 24 h after release for flow cytometry. To block cells in mitosis, nocodazole was added at the 10 h time-point. C. Cdt1-3NLSmyc expressing cells show a higher apoptotic response. Cells treated as in A were cultured for the indicated days, and collected to monitor the cleavage of caspase 3 (Cas-3) by immunoblotting. Cleaved caspase-3 levels in UV-irradiated cells were normalized to those of non-irradiated cells for each cell line.

We then examined the effect of UV irradiation in mitosis in an opposite way by isolating HEK293 cells stably expressing an extra copy of Cdt1-3NLSmyc, HEK293-Cdt1. When this cell line was UV-irradiated during mitosis and released, small amounts of Cdt1 remained in HEK293-Cdt1 cells as compared with HEK293 cells (Fig. 6A). In accordance, more Mcm2 and Mcm6 proteins were detected on the chromatin in HEK293-Cdt1 cells than in control HEK293 cells. Many of the HEK293-Cdt1 cells had entered into S phase (Fig. 6B), whereas HEK293 cells had a high frequency of G1 arrest. Consistently, the level of chromatin-associated PCNA after 12 h was higher in HEK293-Cdt1 cells. Because these cells entered S phase despite having DNA damage, we expected that they were more likely to suffer from replication stress and induced cell death. Thus, we evaluated apoptosis induction based on the cleavage of caspase-3. HEK293 cells and HEK293-Cdt1 cells were exposed to UV irradiation during M phase, and then released and cultured for 1 to 3 days. Immunoblotting revealed higher amounts of cleaved forms of caspase-3 in HEK293-Cdt1 cells (Fig. 6C), suggesting that Cdt1 degradation-dependent G1 arrest facilitates cell survival.

## Discussion

DNA damaging agents induce Cdt1 degradation in G1 phase, however, we found that mitotic cells were resistant to Cdt1 degradation after UV-irradiation. When M-UVIR cells entered G1 phase, Cdt1 was degraded at the end of M phase or in early G1 phase before licensing was established. Cdt1 degradation was dependent on its PIP-box, confirming that CRL4^Cdt2^-mediated proteolysis started operating after release. In these cells, licensing of DNA replication was severely inhibited. Importantly, we noticed that many of these cells were prevented from entering into S phase, in part due to the activation of a DNA damage checkpoint response. In addition, our findings suggested that a replication-licensing checkpoint also contributed to the G1 arrest response of UV-irradiated mitotic cells. First, the extent of G1 arrest was greater in M-UVIR cells than in G1-UVIR cells, although the DNA damage levels and checkpoint activation were almost the same between them. Second, when Cdt1 degradation levels were manipulated by ectopically expressing Cdt2 or Cdt1, the population of G1-arrested cells was affected. In cells expressing an extra-copy of Cdt2, a substrate receptor of CRL4 E3 ligase, Cdt1 degradation was enhanced and thus licensing was severely inhibited. In these cells, the G1 arrest response was greater than that in control cells. On the other hand, cells expressing an extra-copy of Cdt1 had more licensing levels and a smaller G1 arrest response.

The DNA damage response induced during mitosis is not well elucidated. Recent reports demonstrated that upon double-strand break induction by ionizing irradiation in mitotic cells, the cells activated an early DNA damage response, such as activation of ATM, H2AX phosphorylation, and recruitment of the MDC1 and MRN complexes, but recruitment of RNF8, 53BP1, and BRCA1 was inhibited due to phosphorylation by mitotic kinases [35,36,37]. Despite having double-strand breaks, these cells exited mitosis with kinetics similar to those of non-irradiated cells, entered into G1 phase, and exhibited full activation of the DNA damage response. Consequently, these cells underwent G1 cell-cycle arrest. UV irradiation in mitosis did not induce Cdt1 degradation, suggesting that similar to the case after double-strand break induction, the full DNA damage repair pathway might not be operating after UV-irradiation in mitosis. We also found that when cells were treated with another DNA damaging reagent, MMS, Cdt1 was not degraded in M phase, but was degraded after release into G1 phase, similar to UV-irradiated cells, generalizing as a cell response to various types of DNA damage in M phase. Previous data showed that Cdt1 is degraded depending on the activation of the NER process after UV-irradiation. MMS is a base-damaging alkylating agent that is repaired mostly by base excision repair[38]. N-methyl-N-nitro-N-nitrosoguanidine is another alkylating agent that also induces degradation of another CRL4^Cdt2^ target, p21, but depends on mismatch repair proteins[39]. CRL4^Cdt2^-mediated ubiquitination of substrate proteins require chromatin-loaded PCNA. We detected PCNA associated with chromatin after UV-irradiation in mitosis (Fig. 2C, 0 h) and the same levels of PCNA were found at 3 h after release, when Cdt1 was degraded. Although the levels of PCNA detected on chromatin were lower than those detected in S phase, it appeared that the PCNA detected at those time points on chromatin was enough for Cdt1 degradation (Fig. 2C). We speculate that although PCNA was loaded on chromatin during repair of DNA damage, other factors prevented Cdt1 from degrading in M phase. Both Cdt1 and Cdt2 were present in hyperphosphorylated forms in M phase, which might prevent their association with PCNA for Cdt1 degradation. As cells exited M phase, both Cdt1 and Cdt2 were dephosphorylated, and they may have then become ready for ubiquitination.

Although Cdt1 normally functions at the end of mitosis or early G1 phase for replication licensing, it appears that Cdt1 was preferentially recruited to the DNA-damaged sites for destruction rather than to origin-recognition complex-bound sites. This notion suggests that the cell response to DNA damage induced during mitosis is to primarily block licensing by degrading Cdt1. Prevention of replication-licensing could be reinforced by inhibiting the function of Cdt1 through phosphorylation. Previous reports demonstrated that Cdt1 is phosphorylated by mitogen-activated protein (MAP) kinases upon stress induced by treatment with high concentrations of sorbitol, and phosphorylation of Cdt1 inhibits its licensing activity [40,41]. Similarly, UV irradiation activates MAP kinase to phosphorylate and inhibit Cdt1 function. UV irradiation activates checkpoint kinases in G1 phase through the NER pathway, but at much lower levels than in S phase [30,42,43]. Thus, mitotic cells with UV-induced DNA damage may benefit from having a CRL4^Cdt2^ ubiquitin system to degrade Cdt1 and activate the licensing checkpoint. Because mitotic chromosomes must be decondensed for proper chromatin function, including gene transcription in the ensuing cell cycle, activation of a licensing checkpoint would provide enough time for M-UVIR cells to decondense and remodel chromatin in the presence of DNA damage and ongoing repair processes. The importance of the G1 arrest response triggered by the licensing checkpoint of M-UVIR cells was revealed by the cell survival assay. Compared with G1-UVIR cells, M-UVIR cells formed more colonies.

When licensing factors are depleted in yeast cells, they progress into the cell cycle despite the lack of DNA replication taking place, resulting in abortive mitotic cell death [44,45,46]. In contrast, normal mammalian cells possess a licensing checkpoint control [33,34,47,48,49]. When licensing factors, Cdc6, Cdt1, or MCM proteins, are depleted in normal cells, the cell cycle progression of these cells is blocked at the restriction-point [33,48]. Such cells were defective in Cyclin D expression, and in Cdk2 activation due to loss of T160 phosphorylation. In contrast, cancer cells do not show a licensing checkpoint response, as cancer cells depleted of these factors enter into S phase with only a small number of replication origins fired [34]. Eventually, such cancer cells accumulated DNA damage and induced cell death. We used cancer cells, like HeLa and adenovirus-transformed HEK293 cells in the present study and demonstrated that these cells in response to mitotic DNA damage have a defective licensing response that leads to cell cycle arrest in G1 phase, suggesting that licensing defective response is independent of restriction-point regulation. We monitored the phosphorylation levels at T160 of Cdk2, but did not observe a decrease (Fig. 4A). Our results suggest that cells with DNA damaged during mitosis have another type of licensing checkpoint response that might be coupled with a repair process, which operates in such a way that the repair process prevents origin-licensing, and the resulting activation of licensing checkpoint blocks the cells from entering into S phase. On the other hand, it is also possible that even cancer cells possess a small amount of licensing checkpoint response activity compared with normal cells. Despite their low levels, the synergistic effects of the licensing checkpoint control and the DNA damage checkpoint control in cancer cells might contribute to the cell cycle arrest response.

Our finding here can be applied to chemotherapeutic treatment of cancer cells. Many chemotherapeutic agents act to block cell-cycle machineries, including DNA replication and microtubule formation. These drugs, however, also damage normal cells. Given that normal cells possess high licensing checkpoint ability and that DNA damage during mitosis induces different levels of Cdt1 degradation depending on the amount of damage, the combination of mitosis-specific drugs, used at a lower concentration, with DNA-damaging reagents that induce Cdt1 degradation might act to specifically abrogate and kill malignant cells.

## Materials and Methods

### Cell Culture

HeLa cells, HEK293 cells, and U2OS (an osteosarcoma cell line, purchased from ATCC, HTB-96) cells were cultured in Dulbecco’s modified Eagle’s medium with 10% fetal bovine serum and 5% CO_2_. HEK293 cells stably expressing Cdt2-FLAG were described previously [30]. HEK293 cells stably expressing Cdt1-3NLSmyc or PIP-box mutated Cdt1 (A6-Cdt2-3NLSmyc) were isolated after transfection with pCMV-Cdt1-3NLSmyc or pCMV-A6-Cdt1-3NLSmyc [8], respectively. HeLa cells were synchronized in M phase after double thymidine block (first thymidine 2 mM for 15 h, release for 9 h, and second thymidine for 15 h and released in the presence of nocodazole (40 ng/ml) for 9 h; U2OS cells after single thymidine block (2 mM thymidine for 15 h), and released in the presence of nocodazole (40 ng/ml) for 12 h; HEK293 cells after double thymidine block (first thymidine 2 mM for 15 h, release for 8 h, and second thymidine for 15 h) and released in the presence of nocodazole (400 ng/ml) for 12 h). UV-C (254 nm) irradiation of whole cells in dishes was performed in the presence of 5 ml Dulbecco’s modified Eagle’s medium in 10-cm dishes at 20 to 100 J/m^2^ using a UV cross-linker (FS-800, Funakoshi). Methyl methane sulfonate (MMS) was used at 1mM. Flow cytometry analysis of cell cycle was performed as described previously [30].

### Antibodies, Western Blotting, and Immunofluorescence

For Western blotting, whole cell lysates were prepared by lysing cell pellets directly in sodium dodecyl (SDS)-polyacrylamide gel electrophoresis buffer. For immunofluorescence with the Mcm3 antibody, HeLa cells were pre-extracted with phosphate-buffered saline (PBS) containing 0.5% (v/v) Triton X-100, and fixed in 4% paraformaldehyde solution (WAKO) for immunostaining. Images were acquired by Keyence BZ-8100 and nuclear staining intensity was measured by Dynamic Cell count software (Keyence). The following primary antibodies were used: Cdt1[6], Cdt2[50], Mcm2 (lab stock), Mcm3 (ab4460, Abcam, Cambridge, UK), Mcm4 (lab stock), Mcm6 (Santa Cruz Biotechnology, Dallas, TX), Cyclin A (mouse, Ab-6, Neomarkers; rabbit, H-432, Santa Cruz Biotechnology), Cyclin B (H433, Santa Cruz Biotechnology), PCNA (PC10, Santa Cruz Biotechnology), caspase-3 (96625, Santa Cruz Biotechnology), RCC1 (lab stock), Chk1 pS296 (Cell Signaling Technology, Danvers, MA), Chk2 pT68 (Cell Signaling Technology), Cdk2 pT160 (Cell Signaling Technology), and CPD (Cosmo Bio, Tokyo, Japan). Protein levels were analyzed by ImageJ software.

### Chromatin Fractionation

Cell extracts were prepared using 0.1% Triton X-100-containing mCSK buffer (10 mM Pipes, pH 7.9, 100 mM NaCl, 300 mM sucrose, 0.1% [v/v] Triton X-100, 1 mM phenylmethylsulfonyl fluoride, 10 mM ß-glycerophosphate, 1 mM Na_3_VO_4_, 10 mM NaF and 1x protease inhibitor cocktail (Roche)). Approximately 5x10^5^cells were washed with ice-cold PBS and lysed with 0.1 ml of 0.1% Triton X-100 mCSK buffer for 15 min on ice. After centrifugation (15,000 rpm for 15 min at 4^°^C), the precipitate was washed with the same volume of ice-cold 0.1% Triton X-100 mCSK buffer and subsequently suspended in SDS sample buffer.

### Cell survival assay

Mitotic cells and G1 phase cells were UV-irradiated at doses of 25 and 50 J/m2, and cultured for 2 weeks. The cells were washed with PBS, and fixed and stained in a buffer containing 2.5 g/ml Coomassie Brilliant Blue, 7% acetic acid, and 50% methanol, and the colonies were counted.

### Dot blot analysis of CPD

To isolate high molecular-weight DNA, cells were lysed in HMW buffer (10 mM Tris-HCl pH 8.0, 10 mM EDTA, 150 mM NaCl, and 0.1% SDS), incubated in the presence of proteinase K (10 mg/ml) at 55°C for 1 h, and extracted with phenol. DNA was blotted on Hybond-N+ membranes (GE Healthcare), baked, and blotted with anti-CPD antibody.

## References

[1] Nurse P (1994) Ordering S phase and M phase in the cell cycle. Cell 79: 547–550. 7954820 10.1016/0092-8674(94)90539-8

[2] Bell SP, Dutta A (2002) DNA replication in eukaryotic cells. Annu Rev Biochem 71: 333–374. 12045100 10.1146/annurev.biochem.71.110601.135425

[3] Nishitani H, Lygerou Z (2002) Control of DNA replication licensing in a cell cycle. Genes Cells 7: 523–534. 12059957 10.1046/j.1365-2443.2002.00544.x

[4] Blow JJ, Dutta A (2005) Preventing re-replication of chromosomal DNA. Nat Rev Mol Cell Biol 6: 476–486. 15928711 10.1038/nrm1663 PMC2688777

[5] Masai H, Matsumoto S, You Z, Yoshizawa-Sugata N, Oda M (2010) Eukaryotic chromosome DNA replication: where, when, and how? Annu Rev Biochem 79: 89–130. 10.1146/annurev.biochem.052308.103205 20373915

[6] Nishitani H, Taraviras S, Lygerou Z, Nishimoto T (2001) The human licensing factor for DNA replication Cdt1 accumulates in G1 and is destabilized after initiation of S-phase. J Biol Chem 276: 44905–44911. 11555648 10.1074/jbc.M105406200

[7] Arias EE, Walter JC (2007) Strength in numbers: preventing rereplication via multiple mechanisms in eukaryotic cells. Genes Dev 21: 497–518. 17344412 10.1101/gad.1508907

[8] Nishitani H, Sugimoto N, Roukos V, Nakanishi Y, Saijo M, Obuse C, et al (2006) Two E3 ubiquitin ligases, SCF-Skp2 and DDB1-Cul4, target human Cdt1 for proteolysis. Embo J 25: 1126–1136. 16482215 10.1038/sj.emboj.7601002 PMC1409712

[9] Senga T, Sivaprasad U, Zhu W, Park JH, Arias EE, Walter JC, et al (2006) PCNA is a cofactor for Cdt1 degradation by CUL4/DDB1-mediated N-terminal ubiquitination. J Biol Chem 281: 6246–6252. 16407252 10.1074/jbc.M512705200

[10] Abbas T, Dutta A (2011) CRL4Cdt2: master coordinator of cell cycle progression and genome stability. Cell Cycle 10: 241–249. 21212733 10.4161/cc.10.2.14530 PMC3025761

[11] Havens CG, Walter JC (2011) Mechanism of CRL4Cdt2, a PCNA-dependent E3 ubiquitin ligase. Genes Dev 25: 1568–1582. 10.1101/gad.2068611 21828267 PMC3182024

[12] Arias EE, Walter JC (2006) PCNA functions as a molecular platform to trigger Cdt1 destruction and prevent re-replication. Nat Cell Biol 8: 84–90. 16362051 10.1038/ncb1346

[13] Higa LA, Wu M, Ye T, Kobayashi R, Sun H, Zhang H (2006) CUL4-DDB1 ubiquitin ligase interacts with multiple WD40-repeat proteins and regulates histone methylation. Nat Cell Biol 8: 1277–1283. 17041588 10.1038/ncb1490

[14] Jin J, Arias EE, Chen J, Harper JW, Walter JC (2006) A family of diverse Cul4-Ddb1-interacting proteins includes Cdt2, which is required for S phase destruction of the replication factor Cdt1. Mol Cell 23: 709–721. 16949367 10.1016/j.molcel.2006.08.010

[15] Sansam CL, Shepard JL, Lai K, Ianari A, Danielian PS, Amsterdam A, et al (2006) DTL/CDT2 is essential for both CDT1 regulation and the early G2/M checkpoint. Genes Dev 20: 3117–3129. 17085480 10.1101/gad.1482106 PMC1635147

[16] Havens CG, Walter JC (2009) Docking of a specialized PIP Box onto chromatin-bound PCNA creates a degron for the ubiquitin ligase CRL4Cdt2. Mol Cell 35: 93–104. 10.1016/j.molcel.2009.05.012 19595719 PMC2744448

[17] Michishita M, Morimoto A, Ishii T, Komori H, Shiomi Y, Higuchi Y, et al (2011) Positively charged residues located downstream of PIP box, together with TD amino acids within PIP box, are important for CRL4(Cdt2) -mediated proteolysis. Genes Cells 16: 12–22. 10.1111/j.1365-2443.2010.01464.x 21143559

[18] Havens CG, Shobnam N, Guarino E, Centore RC, Zou L, Kearsey, SE, et al. (2012) Direct Role for proliferating cell nuclear antigen (PCNA) in substrate recognition by the E3 Ubiquitin ligase CRL4-Cdt2. J Biol Chem. 10.1074/jbc.M111.337683 PMC3322809 22303007

[19] Harper JW, Elledge SJ (2007) The DNA damage response: ten years after. Mol Cell 28: 739–745. 18082599 10.1016/j.molcel.2007.11.015

[20] Ciccia A, Elledge SJ (2010) The DNA damage response: making it safe to play with knives. Mol Cell 40: 179–204. 10.1016/j.molcel.2010.09.019 20965415 PMC2988877

[21] Sugasawa K, Okamoto T, Shimizu Y, Masutani C, Iwai S, Hanaoka F (2001) A multistep damage recognition mechanism for global genomic nucleotide excision repair. Genes Dev 15: 507–521. 11238373 10.1101/gad.866301 PMC312644

[22] Friedberg EC, Aguilera A, Gellert M, Hanawalt PC, Hays JB, Lehmann AR, et al (2006) DNA repair: from molecular mechanism to human disease. DNA Repair (Amst) 5: 986–996. 16955546 10.1016/j.dnarep.2006.05.005

[23] Gillet LC, Scharer OD (2006) Molecular mechanisms of mammalian global genome nucleotide excision repair. Chem Rev 106: 253–276. 16464005 10.1021/cr040483f

[24] Araujo SJ, Tirode F, Coin F, Pospiech H, Syvaoja JE, Stucki M, et al (2000) Nucleotide excision repair of DNA with recombinant human proteins: definition of the minimal set of factors, active forms of TFIIH, and modulation by CAK. Genes Dev 14: 349–359. 10673506 PMC316364

[25] Ishii T, Shiomi Y, Takami T, Murakami Y, Ohnishi N, Nishitani H (2010) Proliferating cell nuclear antigen-dependent rapid recruitment of Cdt1 and CRL4Cdt2 at DNA-damaged sites after UV irradiation in HeLa cells. J Biol Chem 285: 41993–42000. 10.1074/jbc.M110.161661 20929861 PMC3009925

[26] Shiomi Y, Hayashi A, Ishii T, Shinmyozu K, Nakayama J, Sugasawa K, et al (2012) Two different replication factor C proteins, Ctf18 and RFC1, separately control PCNA-CRL4Cdt2-mediated Cdt1 proteolysis during S phase and following UV irradiation. Mol Cell Biol 32: 2279–2288. 10.1128/MCB.06506-11 22493068 PMC3372265

[27] Raman M, Havens CG, Walter JC, Harper JW (2011) A genome-wide screen identifies p97 as an essential regulator of DNA damage-dependent CDT1 destruction. Mol Cell 44: 72–84. 10.1016/j.molcel.2011.06.036 21981919 PMC3190166

[28] Roukos V, Kinkhabwala A, Colombelli J, Kotsantis P, Taraviras S, Nishitani H, et al (2011) Dynamic recruitment of licensing factor Cdt1 to sites of DNA damage. J Cell Sci 124: 422–434. 10.1242/jcs.074229 21224399

[29] Stathopoulou A, Roukos V, Petropoulou C, Kotsantis P, Karantzelis N, Nishitani H, et al (2012) Cdt1 is differentially targeted for degradation by anticancer chemotherapeutic drugs. PLoS One 7: e34621 10.1371/journal.pone.0034621 22479651 PMC3316709

[30] Sakaguchi H, Takami T, Yasutani Y, Maeda T, Morino M, Ishii T, et al (2012) Checkpoint kinase ATR phosphorylates Cdt2, a substrate receptor of CRL4 ubiquitin ligase, and promotes the degradation of Cdt1 following UV irradiation. PLoS One 7: e46480 10.1371/journal.pone.0046480 23029527 PMC3460910

[31] Ralph E, Boye E, Kearsey SE (2006) DNA damage induces Cdt1 proteolysis in fission yeast through a pathway dependent on Cdt2 and Ddb1. EMBO Rep 7: 1134–1139. 17039252 10.1038/sj.embor.7400827 PMC1679788

[32] Morino M, Tanaka M, Shiomi Y, Nishitani H (2014) Imaging analysis to determine chromatin binding of the licensing factor MCM2-7 in mammalian cells. Methods Mol Biol 1170: 529–537. 10.1007/978-1-4939-0888-2_29 24906334

[33] Nevis KR, Cordeiro-Stone M, Cook JG (2009) Origin licensing and p53 status regulate Cdk2 activity during G(1). Cell Cycle 8: 1952–1963. 19440053 10.4161/cc.8.12.8811 PMC2972510

[34] Lau E, Chiang GG, Abraham RT, Jiang W (2009) Divergent S phase checkpoint activation arising from prereplicative complex deficiency controls cell survival. Mol Biol Cell 20: 3953–3964. 10.1091/mbc.E09-01-0022 19587119 PMC2735493

[35] Giunta S, Belotserkovskaya R, Jackson SP (2010) DNA damage signaling in response to double-strand breaks during mitosis. J Cell Biol 190: 197–207. 10.1083/jcb.200911156 20660628 PMC2930281

[36] Orthwein A, Fradet-Turcotte A, Noordermeer SM, Canny MD, Brun CM, Strecker J, et al (2014) Mitosis inhibits DNA double-strand break repair to guard against telomere fusions. Science 344: 189–193. 10.1126/science.1248024 24652939

[37] Peterson SE, Li Y, Chait BT, Gottesman ME, Baer R, Gautier J (2011) Cdk1 uncouples CtIP-dependent resection and Rad51 filament formation during M-phase double-strand break repair. J Cell Biol 194: 705–720. 10.1083/jcb.201103103 21893598 PMC3171114

[38] Seo YR, Fishel ML, Amundson S, Kelley MR, Smith ML (2002) Implication of p53 in base excision DNA repair: in vivo evidence. Oncogene 21: 731–737. 11850801 10.1038/sj.onc.1205129

[39] Jascur T, Fotedar R, Greene S, Hotchkiss E, Boland CR (2011) N-methyl-N'-nitro-N-nitrosoguanidine (MNNG) triggers MSH2 and Cdt2 protein-dependent degradation of the cell cycle and mismatch repair (MMR) inhibitor protein p21Waf1/Cip1. J Biol Chem 286: 29531–29539. 10.1074/jbc.M111.221341 21725088 PMC3190993

[40] Chandrasekaran S, Tan TX, Hall JR, Cook JG (2011) Stress-stimulated mitogen-activated protein kinases control the stability and activity of the Cdt1 DNA replication licensing factor. Mol Cell Biol 31: 4405–4416. 10.1128/MCB.06163-11 21930785 PMC3209262

[41] Miotto B, Struhl K (2011) JNK1 phosphorylation of Cdt1 inhibits recruitment of HBO1 histone acetylase and blocks replication licensing in response to stress. Mol Cell 44: 62–71. 10.1016/j.molcel.2011.06.021 21856198 PMC3190045

[42] Marti TM, Hefner E, Feeney L, Natale V, Cleaver JE (2006) H2AX phosphorylation within the G1 phase after UV irradiation depends on nucleotide excision repair and not DNA double-strand breaks. Proc Natl Acad Sci U S A 103: 9891–9896. 16788066 10.1073/pnas.0603779103 PMC1502549

[43] Marini F, Nardo T, Giannattasio M, Minuzzo M, Stefanini M, Plevani P (2006) DNA nucleotide excision repair-dependent signaling to checkpoint activation. Proc Natl Acad Sci U S A 103: 17325–17330. 17088560 10.1073/pnas.0605446103 PMC1859929

[44] Kelly TJ, Martin GS, Forsburg SL, Stephen RJ, Russo A, Nurse P (1993) The fission yeast cdc18+ gene product couples S phase to START and mitosis. Cell 74: 371–382. 7916658 10.1016/0092-8674(93)90427-r

[45] Piatti S, Lengauer C, Nasmyth K (1995) Cdc6 is an unstable protein whose de novo synthesis in G1 is important for the onset of S phase and for preventing a 'reductional' anaphase in the budding yeast Saccharomyces cerevisiae. EMBO J 14: 3788–3799. 7641697 10.1002/j.1460-2075.1995.tb00048.x PMC394453

[46] Nishitani H, Lygerou Z, Nishimoto T, Nurse P (2000) The Cdt1 protein is required to license DNA for replication in fission yeast. Nature 404: 625–628. 10766248 10.1038/35007110

[47] Shreeram S, Sparks A, Lane DP, Blow JJ (2002) Cell type-specific responses of human cells to inhibition of replication licensing. Oncogene 21: 6624–6632. 12242660 10.1038/sj.onc.1205910 PMC3605503

[48] Liu P, Slater DM, Lenburg M, Nevis K, Cook JG, Vaziri C (2009) Replication licensing promotes cyclin D1 expression and G1 progression in untransformed human cells. Cell Cycle 8: 125–136. 19106611 10.4161/cc.8.1.7528 PMC3032797

[49] Feng H, Kipreos ET (2003) Preventing DNA re-replication—divergent safeguards in yeast and metazoa. Cell Cycle 2: 431–434. 12963835

[50] Nishitani H, Shiomi Y, Iida H, Michishita M, Takami T, Tsurimoto T (2008) CDK inhibitor p21 is degraded by a proliferating cell nuclear antigen-coupled Cul4-DDB1Cdt2 pathway during S phase and after UV irradiation. J Biol Chem 283: 29045–29052. 10.1074/jbc.M806045200 18703516 PMC2662008

